# Use of pharmaceuticals amongst athletes tested by Anti-Doping Norway in a five-year period

**DOI:** 10.3389/fspor.2023.1260806

**Published:** 2023-10-04

**Authors:** Astrid Gjelstad, Tine Marie Herlofsen, Anne-Linn Bjerke, Fredrik Lauritzen, Ingunn Björnsdottir

**Affiliations:** ^1^ Science and Medicine, Anti-Doping Norway, Oslo, Norway; ^2^ Department of Pharmaceutical Chemistry, School of Pharmacy, University of Oslo, Oslo, Norway; ^3^ Department of Pharmaceutics and Social Pharmacy, School of Pharmacy, University of Oslo, Oslo, Norway

**Keywords:** pharmaceuticals, sport, ATC system, doping control forms, anti-doping

## Abstract

**Introduction:**

The aim of the study was to map the use of pharmaceuticals by Norwegian athletes registered on doping control forms (DCFs) in a five-year period to examine general and some class specific use of pharmaceuticals across sports and athlete levels.

**Method:**

Anonymous data from DCFs collected in 2015-2019 were manually entered into a database using the Anatomical Therapeutic Chemical (ATC) system for classification of the pharmaceuticals. Variables entered were year of control, gender, age group, athlete level, sport, test type, nationality, and pharmaceuticals (and dietary supplements) used.

**Results:**

Pain killers in the ATC groups M01 A (Nonsteroidal anti-inflammatory drugs - NSAIDs) and N02 B (other analgesics), and anti-asthmatics in ATC groups R03 A and R03 B were the most frequently used pharmaceuticals. National level athletes reported more use of pharmaceuticals (1.4 ± 1.7 pharmaceuticals per form) than recreational level athletes (0.9 ± 1.2). The highest proportion of DCFs containing information about at least one pharmaceutical were found in speed skating (79.1%), alpine skiing (74.0%), rowing (72.4%) and cross-country skiing (71.7%). Painkillers were most frequently used in muscular endurance sports (30.4% and 21.2 % for M01A and N02 B, respectively) and ball and team sports (17.9% and 17.0%). Use of hypnotics was reported from ice-hockey players and alpine skiers in around 8% of the cases.

**Coclusion:**

Use of anti-asthmatics was most often reported amongst athletes specially exposed to cold, chemicals and heavy endurance training. Athletes in specialized sports requiring high levels of strength and/or endurance reported a higher use of pharmaceuticals out-of-competition compared to in-competition, while there was no such difference in complex sports, such as team, gymnastic, aiming and combat sports.

## Introduction

Athletes, like everyone else, on occasion need treatment with pharmaceuticals, for chronic or acute conditions. Since the use of pharmaceuticals is normally linked to symptoms and diseases, athletes, often portrayed as embodiments of a healthy lifestyle, are thus expected to have a relatively low consumption of pharmaceuticals. Conversely, athletes are also often exposed to extreme stress during training and competition, which might lead to increased use of pharmaceuticals.

The World Anti-Doping Agency (WADA) annually publishes a list of substances and methods that are prohibited in sports (1). Many of these substances are found as Active Pharmaceutical Ingredients (APIs) in legal pharmaceuticals. Of all registered pharmaceuticals in The Norwegian Pharmaceutical Product Compendium (2), approximately 13% involve substances or methods prohibited by WADA. Being vigilant when using pharmaceuticals should be a part of active athletes' everyday life in order to avoid unintentional doping, similarly, as has been reported for dietary supplements [e.g., (3)]. The use of pharmaceuticals is thus a part of the athletes' exposome (4), and should also form a part of the athletes' health literacy (5, 6).

All pharmaceutical substances are classified in the Anatomic Therapeutic Chemical (ATC) classification system by The WHO collaborating center for drug statistics methodology (7). The system is intended for monitoring development in usage of pharmaceuticals and for comparison and is widely used for those purposes. It could also potentially be useful in classification of athletes' use of pharmaceuticals. Various studies have examined the use of pharmaceuticals in sports (8–10), within different sport disciplines (11–13), nationalities (14, 15), age groups (16), during major sports events (14, 17, 18) or with focus on specific pharmacological classes, e.g., analgesics (19–21), antibiotics (22, 23), anti-asthmatics (24), allergy medication (25) and decongestants (26). A challenge with the existing literature is the use of different methodologies to map and study the use of pharmaceuticals, making comparisons difficult. By using the ATC system for classification of pharmaceuticals in sport, data for pharmaceuticals down to substance level (fifth level) or pharmacological or therapeutic subgroup (third level) would be readily available.

A better knowledge about the use of pharmaceuticals in different sport disciplines is of high relevance for athletes, medical support personnel, and for anti-doping organizations who educate the athletes in correct use of pharmaceuticals and supplements to avoid unintentional doping. Furthermore, frequency and type of pharmaceuticals used in the different disciplines are essential knowledge for discussing a potential medicalization of sport. Claims about such medicalization appear regularly (27, 28). Several studies have reported athletes using painkillers before a competition to avoid possible pain from injuries (20), taking sleeping pills to cope with a tight match program or travel across time zones (29, 30) and using oral antibiotics liberally (8). By focusing on use of pharmaceuticals in sport, athletes and support personnel might be made aware of the potential harmful effects of incorrect use leading to more rational use and better health. Patterns of use of pharmaceuticals can also be a useful source of information for sports pharmacists in their assistance for athletes (31).

In this project, the use of pharmaceuticals by Norwegian athletes registered on doping control forms (DCFs) in a five-year period (2015–2019) is mapped to examine general and some class specific use of pharmaceuticals amongst athletes in Norway.

## Method

Doping control forms (DCFs) from all doping controls where Anti-Doping Norway (ADNO) had been the Testing authority (i.e., the anti-doping organization that authorized testing on athletes it has authority over) from the period 2015–2019 were included in the study. The material included DCFs from doping controls of Norwegian athletes performing their sport in Norway or abroad, as well as from athletes from other countries exercising their sport in Norway under the jurisdiction of a Norwegian sport federation organized under the Norwegian confederation of sport, and Olympic and Paralympic Committee.

The DCFs included in the present study were collected from ADNOs own paper archives, stored according to Norwegian laws and in compliance with World Anti-Doping Code article 14.6 (32). Patients or the public were not involved in the design, or conduct, or reporting, or dissemination plans of our research.

The study was approved by Regional Committees for Medical Research Ethics, ID ​29318. As the data from the Doping Control Forms were fully anonymized, there was no need for the athletes to give informed consent.

### Development of database

The following information from each DCF were manually registered into an electronic database using Microsoft Excel: Year of the control, gender, age group (<20; 20–24; 25–29; 30–34; 35–40; >40), athlete level (i.e., recreational level (RL) or national level athlete (NLA)), sport, sport discipline, type of test (i.e., whether the sample was collected in-competition (the period commencing at 11:59 p.m. on the day before a competition in which the athlete is scheduled to participate through the end of such competition and the sample collection process related to such competition) or out-of-competition) and nationality. Pharmaceuticals and dietary supplements noted by the athlete on the DCF were also recorded, as the athlete is required to register any pharmaceuticals and supplements taken the last seven days before the control. The data were registered fully anonymized, thus no identifiers like name, address, sample code and exact age or date of the control were included in the database.

To compare the use of pharmaceuticals across sport categories, sport disciplines were classified based on physiological characteristics and put into the following groups: Aiming sports, Ball and team sports, Combat sport, Gymnastic sports, Muscular Endurance sports, VO_2_max endurance sports, and Strength and Power sports (33) (see Table 2 for examples of sport disciplines in each category).

The number of DCFs retrieved from the paper archives per year was checked against the WADA Anti-Doping Administration and Management System (ADAMS) DCF report to ensure that the DCF paper copies reflected the actual number of doping tests performed in 2015–2019.

The pharmaceuticals were classified according to the Anatomical Therapeutic Chemical (ATC) Classification System. To easily associate the name of the pharmaceutical product with the ATC code, a matrix was developed in Excel; a list of all registered pharmaceutical products in Norway was obtained from The Norwegian Pharmaceutical Product Compendium (7,510 unique products per 11.06.2020). When a pharmaceutical product name was entered in one cell in Excel, the corresponding ATC code then appeared automatically in the neighbor cell.

The ATC codes were registered down to the substance level (level 5). However, for practical reasons, the ATC codes were recoded to the third level indicating the therapeutic/pharmacological subgroup and most of the data in this paper refers to the third level.

### Statistics

IBM SPSS Statistics v. 28.0.0.0 were used for descriptive statistics. Continuous data and categorical variables are presented as mean ± SD and percentages, respectively. Pearson's chi-square tests were used to test associations between categorical variables. Analysis of variance (ANOVA) was used to compare trends in pharmaceutical use over time. The Mann-Whitney U Test were used to compare the numbers of pharmaceuticals between athlete levels. The threshold for statistical significance was set to *p* = 0.05.

## Results

### General population

Totally 10 418 DCFs (males: 76.2%, *n* = 7,939; females: 23.8%, *n* = 2,479) were included in the study. On average, 2,084 ± 96 annual number of DCFs were recorded from the period 2015–2019. Of the 10 418 DCFs registered, 40.6% of the forms were obtained from recreational athletes (*n* = 4,234), while 59.4% were national or international level athletes (*n* = 6,184) (Table 1). The majority of the athletes were in the age groups 20–24 (34.7%) and 25–29 years (32.2%). Athletes with Norwegian citizenship constituted 93.6% of the DCFs, while 6.4% were from athletes with other nationalities. In total, the material represents athletes from 67 different sport disciplines, where 25 of them encompass more than 100 DCFs each for the whole study period (Table 1). Of all DCFs, 45.6% were collected in-competition (IC), whereas 54.4% were obtained from athletes out-of-competition (OOC) (Table 2).

**Table 1:** Number of doping control forms (DCFs) (*n*) in each sport discipline and athlete level (RA: recreational athlete; NLA: national level athlete)—and proportion of the DCFs containing information about ≥one pharmaceutical (NA: not applicable).

** **	Athlete level				Total	
	RA		NLA		** **	
	*n*	DCFs with ≥ one pharmaceutical	*n*	DCFs with ≥ one pharmaceutical	*n*	DCFs with ≥ one pharmaceutical
Football	436	46.8%	922	48.5%	1,358	47.9%
Cross-country skiing	160	51.9%	829	75.5%	989	71.7%
Cycling	175	56.6%	560	69.8%	735	66.7%
Athletics	207	54.6%	488	65.8%	695	62.4%
Ice hockey	464	46.3%	193	67.4%	657	52.5%
Powerlifting	399	49.4%	250	49.2%	649	49.3%
Handball	260	41.2%	354	55.1%	614	49.2%
Basketball	333	39.3%	47	23.4%	380	37.4%
Biathlon	20	60.0%	358	69.0%	378	68.5%
Weightlifting	161	54.0%	214	52.3%	375	53.1%
Volleyball	141	51.8%	157	47.1%	298	49.3%
American football	209	48.3%	42	47.6%	251	48.2%
Speed skating	12	50.0%	237	80.6%	249	79.1%
Swimming	72	52.8%	172	70.3%	244	65.2%
Floorball	87	46.0%	103	47.6%	190	46.8%
Kickboxing	113	47.8%	77	32.5%	190	41.6%
Triathlon	81	54.3%	92	46.7%	173	50.3%
Boxing	97	32.0%	69	21.7%	166	27.7%
Cheerleading	152	65.1%	<5	NA	154	64.9%
Rowing	22	36.4%	130	78.5%	152	72.4%
Alpine skiing	<5	NA	147	75.5%	150	74.0%
Wrestling	48	39.6%	89	58.4%	137	51.8%
Orienteering	36	47.2%	99	65.7%	135	60.7%
Taekwondo	42	40.5%	63	58.7%	105	51.4%
Judo	71	52.1%	31	67.7%	102	56.9%
Canoe	15	33.3%	80	60.0%	95	55.8%
Nordic combined	<5	NA	83	59.0%	83	59.0%
Karate	45	44.4%	35	57.1%	80	50.0%
Rugby	46	45.7%	12	25.0%	58	41.4%
Air sport	48	62.5%	<5	NA	49	63.3%
Gymnastics	<5	NA	45	35.6%	48	39.6%
Golf	38	55.3%	8	37.5%	46	52.2%
Motorbike	14	42.9%	28	53.6%	42	50.0%
Ski jump	<5	33.3%	35	54.3%	38	52.6%
Shooting	22	50.0%	14	42.9%	36	47.2%
Functional fitness	34	61.8%	<5	NA	34	61.8%
Armwrestling	31	38.7%	<5	NA	34	35.3%
Tennis	11	54.5%	17	52.9%	28	53.6%
Bandy	16	25.0%	11	27.3%	27	25.9%
Sailing	<5	NA	16	68.8%	19	63.2%
Fencing	9	33.3%	9	44.4%	18	38.9%
Snowboard	7	28.6%	7	42.9%	14	35.7%
Table tennis	5	20.0%	8	37.5%	13	30.8%
Dance sport	12	75.0%	<5	0.0%	12	75.0%
Equestrian	6	33.3%	<5	NA	10	40.0%
Company sports	9	33.3%	<5	NA	9	33.3%
Curling	<5	NA	9	66.7%	9	66.7%
Badminton	<5	NA	8	62.5%	8	62.5%
Climbing	<5	NA	<5	NA	8	50.0%
Fitness center	8	37.5%	<5	0.0%	8	37.5%
Billiard	7	57.1%	<5	0.0%	7	57.1%
Telemark	<5	NA	7	42.9%	7	42.9%
Bobsleigh & skeleton	<5	NA	<5	NA	5	NA
Underwater sport	5	NA	<5	NA	5	NA
Baseball	<5	NA	<5	NA	<5	NA
Bowling	<5	NA	<5	NA	<5	NA
Cricket	<5	NA	<5	NA	<5	NA
Freeski	<5	NA	<5	NA	<5	NA
Sleddog	<5	NA	<5	NA	<5	NA
Squash	<5	NA	<5	NA	<5	NA
Waterski	<5	NA	<5	NA	<5	NA
Others	<5	NA	<5	NA	<5	NA
Frisbee	<5	NA	<5	NA	<5	NA
Lacrosse	<5	NA	<5	NA	<5	NA
Archery	<5	NA	<5	NA	<5	NA
Casting	<5	NA	<5	NA	<5	NA
Jujutsu	<5	NA	<5	NA	<5	NA
Total	4,234	48.1%	6,184	61.0%	10,418	55.7%

**Table 2:** Categorization of sport disciplines with associated total number of doping control forms (DCFs); number and proportion of DCFs with information aboutf ≥1 pharmaceutical divided into type of test (sampling out-of-competition (OOC) or in-competition (IC)).

	** **	DCFs			DCFs with pharmaceuticals*			** **
Sport category	Examples of sport disciplines	*n_Total_*	*n*_OOC_ (%)	*n*_IC_ (%)	*n*_Total_	*n*_OOC_ (%)	*n*_IC_ (%)	Pearson Chi-Square *p*-value
Ball and team sports	Basketball, football, handball etc.	4,057	1,412 (34.8%)	2,645 (65.2%)	1,960 (48.3%)	673 (47.7%)	1,287 (48.7%)	0.546
VO_2_max endurance sports	Biathlon, canoe, cross-country skiing, cycling, rowing etc.	3,233	2,381 (73.6%)	852 (26.4%)	2,195 (67.9%)	1,704 (71.6%)	491 (57.6%)	<0.001*
Strength and power sports	Athletics sprint and throws, powerlifting, weightlifting etc.	1,758	1,115 (63.4%)	643 (36.6%)	968 (55.1%)	634 (56.9%)	334 (51.9%)	0.046*
Combat sports	Boxing, fencing, judo, karate, wrestling etc.	799	499 (62.5%)	300 (27.3%)	356 (44.6%)	217 (43.5%)	139 (46.3%)	0.433
Muscular endurance sports	Alpine skiing, climbing, sailing etc.	184	151 (82.1%)	33 (17.9%)	130 (70.7%)	113 (74.8%)	17 (51.5%)	0.008*
Other sports	Air sports, motor sport, underwater sports etc.	151	11 (7.3%)	140 (92.7%)	83 (55.0%)	3 (27.3%)	80 (57.1%)	0.055
Gymnastic sports	Dancing, Gymnastics, ski jump, snowboard etc.	130	81 (62.3%)	49 (37.7%)	61 (46.9%)	39 (48.1%)	22 (44.9%)	0.719
Aiming sports	Archery, curling, golf, shooting etc.	106	17 (16.0%)	89 (84.0%)	54 (50.9%)	10 (58.8%)	44 (49.4%)	0.478
Total		10 418	5,667 (54.4%)	4,751 (45.6%)	5,807 (55.7%)	3,393 (59.9%)	2,414 (50.8%)	<0.001

The Pearson Chi-Square *p*-value indicates whether the difference between *n*_OOC_ and *n*_IC_ within the “DCFs with pharmaceuticals” is significant.

* *p*-value < 0.05 indicates statistically significant difference.

Of all DCFs, 77.7% (*n* = 8,091) contained information about at least one product, that is either a pharmaceutical and/or a dietary supplement, whereof 3,012 forms (28.9%) included both a pharmaceutical and a dietary supplement. Overall, the average number of products per form was 2.4 ± 2.3, whereas the maximum number of products given on a single form was 22. Information about one or more pharmaceuticals were provided on 55.7% (*n* = 5,807) of the DCFs (1.2 ± 1.6), with a maximum of 15. The reported use of dietary supplements is further described in a separate paper (34).

### Trends over time

The proportion of DCFs containing information about one or more pharmaceutical(s) was used as an indicator of general pharmaceutical use. In 2015, the percentage was 52.0% for all DCFs, while the highest percentage was registered in 2019 (58.4%). The difference between 2015 and 2019 is significant (*p* < 0.001) although a decrease was observed in 2018.

**Figure 1:**
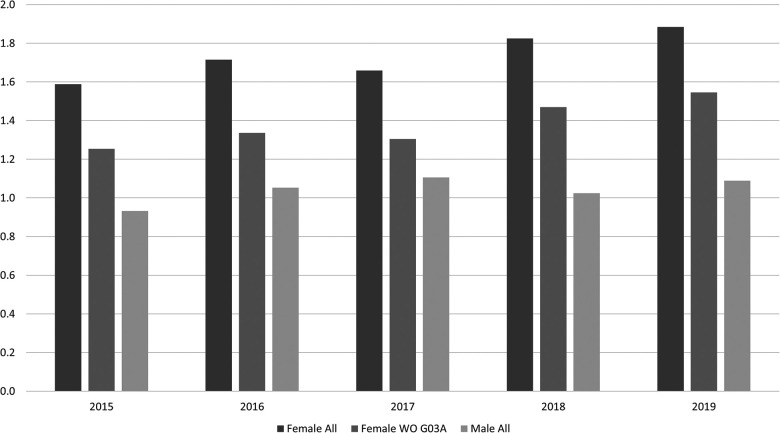
Mean number of pharmaceuticals per doping control form (DCF) from female athletes including (female All) and excluding contraceptives (without (WO) G03A) compared to male athletes (male All) in the period 2015-2019.

A difference between males and females appeared, as regards the degree of increase over time. In the period 2015–2019, the percentage of DFCs with one or more pharmaceutical were relatively constant around 50% for males with no significant differences within the years, whereas the DFCs obtained from female athletes increased significantly from 66.9% to 79.4% in the same period (*p* < 0.001). The mean of pharmaceuticals per DCFs also differed between the genders, even if the oral contraceptives are removed from the results (Figure 1).

### Overall registration of pharmaceuticals

**Figure 2:**
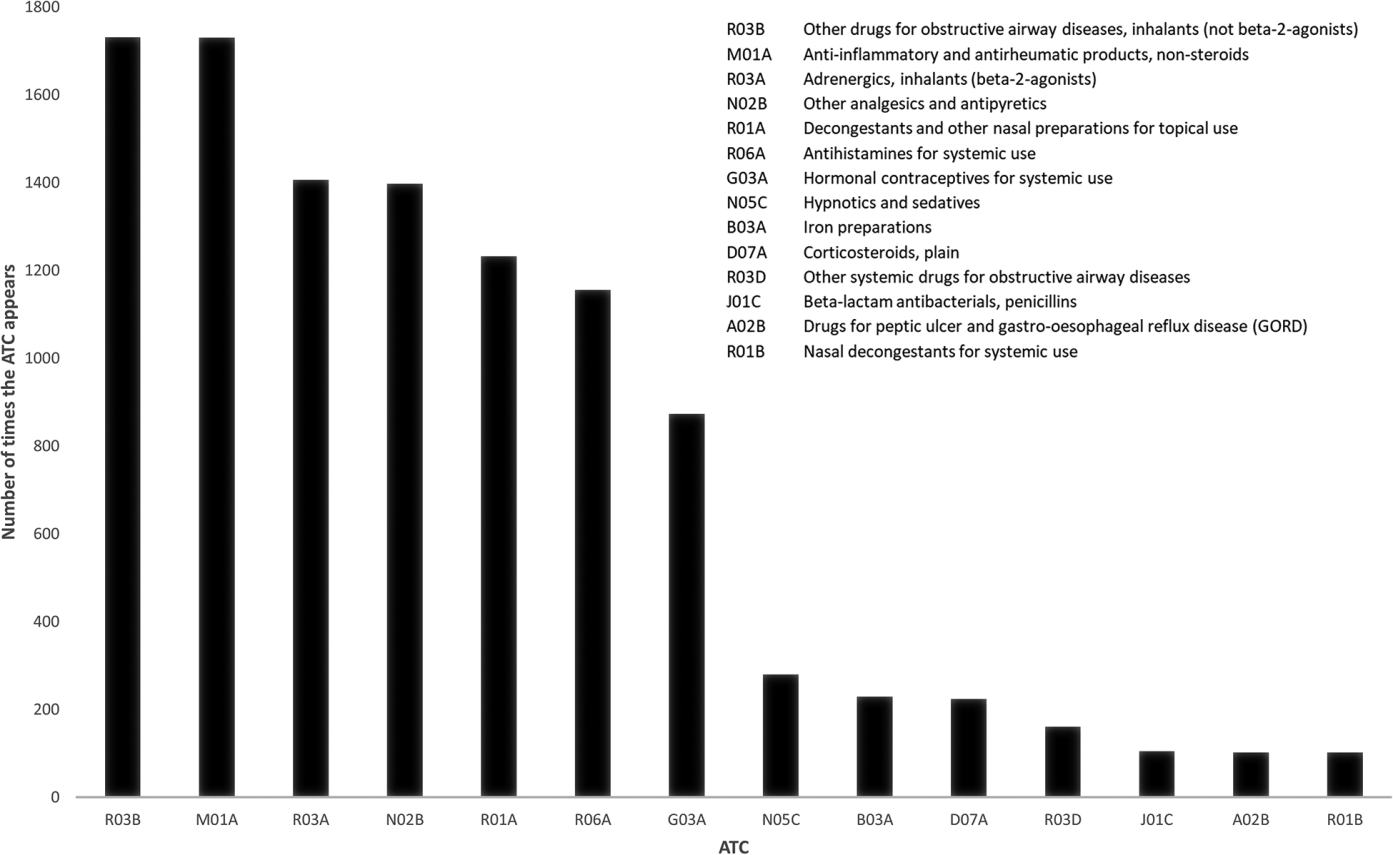
Frequencies of ATC codes (level 3) found on DCFs from the period 2015-2019. Only ATC codes appearing >100 times are included.

Pharmaceuticals representing 126 different ATC codes at level 3 were identified. The most frequent ATC codes appearing in the database were R03B (*Other drugs for obstructive airway diseases, inhalants*) and M01 A (*Anti-inflammatory and antirheumatic products, non-steroids*), which were registered 1,731 and 1,730 times, respectively (on average at 16.6% of all DCFs) (Figure 2). Other pharmaceuticals mainly used in treatment of asthma, ATC code R03 A (*Adrenergics, inhalants*) constitute the third most frequent ATC code (13.5%), closely followed by *Other analgesics and antipyretics* (N02 B), which were found 1,397 times (13.4%). In total, 14 unique level 3 ATC codes appeared in the database 100 times or more (Figure 2).

### Athlete level

The average number of pharmaceuticals registered per DCF was higher amongst national level athletes (NLA) (1.4 ± 1.7) compared to recreational athletes (RA) (0.9 ± 1.2) (*p* < 0.001). The highest number of pharmaceuticals registered on a single DCF was 15 in the NLA group, and eight in the RA group. In 82 cases (1.9%), five or more pharmaceuticals were registered on a single DCF from RA, whereas the corresponding number was 360 (5.8%) from NLA. The same trend appeared when the results were further divided into genders. Female RA declared in average 1.46 ± 1.5 pharmaceuticals per DCFs, while female NLA declared in average 1.88 ± 1.8 pharmaceuticals. The corresponding results for male RA were 0.76 ± 1.1 and 1.26 ± 1.65 for the male NLA, respectively.

### Sport type

Athletes from various sport disciplines display various use of pharmaceuticals (Table 1). The highest share of pharmaceutical use was found among speed skaters (79.1% (197 out of 249) of the DCFs contained information about at least one pharmaceutical), whereas the lowest use was found in boxing (27.7%; 46 out of 166). In general, endurance sport requiring a high VO_2_max used more pharmaceuticals than team- and ball sports.

The data was further split in sport categories for the top four of the most frequently used ATC codes, which include two groups of asthma medication (R01A and R01B) and to groups of pain killers (M01A and N02B), respectively (Table 3). In addition, the results for the hypnotics in ATC group N05C were included in the results, as pharmaceuticals within this ATC group appeared to be frequently used in certain sports. The differences between the sport categories regards to use of asthma medication range from 28.4% of R03B in the VO_2_max endurance sports down to 1.1% within the Ball and team sports. The use of pain killers did not follow a clear pattern between the sport categories; however, muscular endurance athletes tend to use slightly more than others.

**Table 3:** Five of the most frequent ATC codes (level 3) per sport category. The numbers refer to proportion of the doping control forms containing information about ≥one of the respective ATC codes (R03A Adrenergics, inhalants (beta-2-agonists); R03B Other drugs for obstructive airway diseases, inhalants (not beta-2-agonists); M01A Anti-inflammatory and antirheumatic products, non-steroidal; N02B Other analgesics and antipyretics; N05C Hypnotics and sedatives).

Asthma			Painkillers			Hypnotics	
*Sport category*	*R03A*	*R03B*	*Sport category*	*M01A*	*N02B*	*Sport category*	*N05C*
VO_2_max endurance sports	22.4%	28.4%	Muscular endurance sports	30.4%	21.2%	Muscular endurance sports	6.5%
Combat sports	7.0%	2.6%	Ball and teams sports	17.9%	17.0%	VO_2_max endurance sports	3.4%
Strength and power sports	5.5%	5.9%	Strength and power sports	17.0%	12.9%	Aiming sports	2.8%
Muscular endurance sports	5.4%	17.4%	Gymnastics sports	16.9%	14.6%	Strength and power sports	2.2%
Ball and teams sports	5.2%	1.1%	Aiming sports	16.0%	12.3%	Ball and teams sports	2.2%
Gymnastics sports	3.8%	1.5%	Fighting sports	10.8%	11.9%	Fighting sports	1.1%
Aiming sports	2.8%	1.9%	VO_2_max endurance sports	9.6%	8.5%	Gymnastics sports	0.0%
Other	4.6%	1.3%	Other	18.5%	17.9%	Other	2.0%
Total	10.7%	10.8%	Total	14.8%	13.3%	Total	2.5%

Use of hypnotics ranges from 6.5% in the muscular endurance sport category to 0.0% in the gymnastic sport. The two sport disciplines ice hockey and alpine skiing differ from the other disciplines, were the proportion of DCFs with at least one hypnotic in ATC group N05C were 8.2% and 8.0%, respectively.

### Ages, genders and type of test

**Figure 3:**
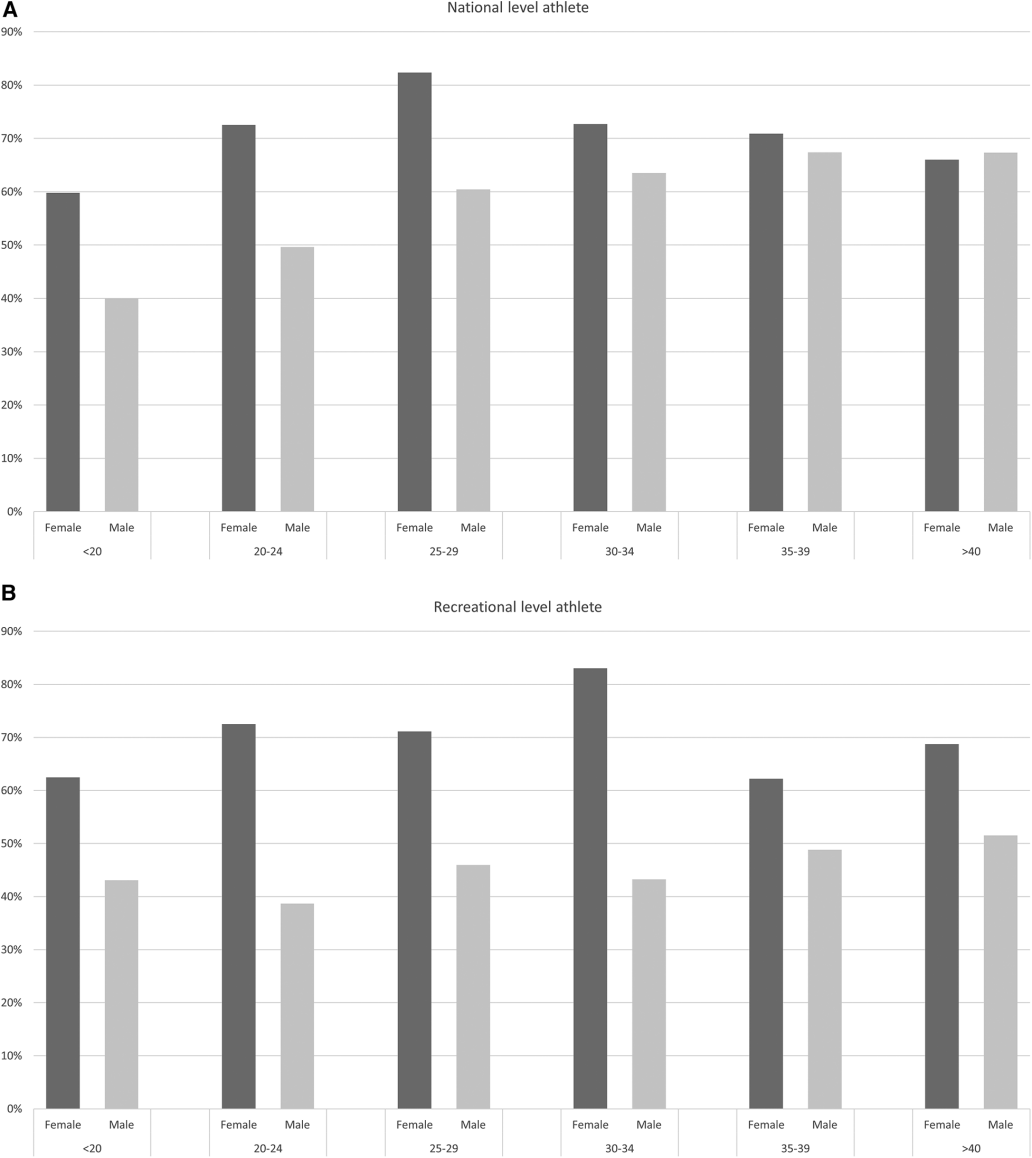
Proportion of doping control forms (DCFs) within age group and gender containing information about ≥one pharmaceutical for (**A**) national level athletes and (**B**) recreational level athletes, respectively.

A peak in the proportion of one or more pharmaceuticals appeared in the age group 25–29 for female national level athletes, with 83% of the DCFs including at least one pharmaceutical (Figure 3). Within the group of female recreational athletes, most pharmaceuticals were used in the group 30–34 years, with information of pharmaceuticals in 82% of the DCFs. For males, there were a slight increase in use of pharmaceuticals with age in both athlete level groups.

For athletes in Ball and team sport, gymnastic sports, aiming sports and combat sports, there were no significant differences in the use of pharmaceuticals IC vs. OOC (Table 2). In contrast, athletes in VO_2_max endurance sports, Strength and Power sports and Muscular endurance sports used significantly more pharmaceuticals OOC vs. IC.

## Discussion

The study examines the use of pharmaceuticals amongst recreational and national level athletes who participated in doping controls performed by ADNO in the period 2015–2019 as part of the national testing program in Norwegian sport. As the focus of this paper is to get a general overview of pharmaceutical usage based on doping control forms, a thorough comparison of the results with existing literature will not be done. Only some examples are given in the following discussion.

### Differences in use of pharmaceuticals

The general trend in use of pharmaceuticals in the period was a slight increase, reflected by an increase in the portion of DCFs containing at least one pharmaceutical from 52.0% in 2015 to 58.4% in 2019. The trend is likely to reflect a general trend of increased use of pharmaceuticals in Norway in the period 2015–2019, even if the indicators are not directly comparable, i.e., number of DCFs vs. Defined Daily Dosages (DDD)) (35). The increasing trend in use of pharmaceuticals is also detected in sport in some reports (10, 36), while no significant increased trend was found in the football World Cups between 2002 and 2014 (21).

National level athletes tended to use a higher number of pharmaceuticals than recreational level athletes. As many NLA are professional athletes, they in most cases ten exercise more and harder than recreational athletes, thus, being more prone to extreme stress that can lead to injuries and other conditions that require pharmaceutical intervention, e.g., by painkillers or anti-asthmatics (8, 37, 38).

Pharmaceuticals were listed on a relatively high proportion of DCFs from female athletes compared to males. The difference in use of pharmaceuticals between men and women in the general population is well-known and reflected both in the national reports from the same period (35) and in the scientific literature (39–41). Differences in use of pharmaceuticals between men and women in sport is also reported (42, 43). The results from the present study are therefore in line with literature. Even if the ATC code G03A (i.e., hormonal contraceptives for systemic use) is excluded from the calculations, women report a higher mean number of pharmaceutical per DCF than men. A possible explanation is a more frequent doctor consultations amongst females, more seeking of preventive care, and differences in physicians' prescription of pharmaceuticals (44). Gender-related morbidity could also be a reason for the gender differences. For example, asthma incidence differs according to gender (45), as do use of pain killers (46). Since anti-asthmatics and pain killers are among the most frequently used pharmaceuticals in the present study, it could be one of the reasons why differences between gender were observed.

### ATC codes

To our knowledge, no other mapping of pharmaceuticals in sport of comparable magnitude has used ATC codes as a classification system. However, the findings presented here are in line with other studies focusing on medication in sport, revealing high consumption of anti-asthmatics, NSAIDs and other pain killers and nasal preparations (8, 10, 13, 15, 42).

Anti-asthmatics (R03 A and R03 B) and pain killers (M01 A and N02 B) were the most reported pharmaceuticals in the present study. High prevalence of asthma medication is not very surprising, as strenuous aerobic exercise has the potential to induce asthma and thus the requirement of anti-asthmatics (47). This is particularly evident in sports which require constant physical exertion (e.g., triathlon, cycling, rowing), take place in cold weather (cross-country, biathlon, speed skating) or in environments with poor air quality (swimmers in chlorine swimming pools) (48). The same pattern appears in the presented data material, as the VO_2_max endurance sports tops the prevalence list of anti-asthmatics. The data suggests that there are some differences between sport types regarding the use of pharmaceutical IC vs. OOC. Interestingly, sports characterized by performances based on a few bio motor physiological skills rather technical and tactical skills, such as VO_2_max endurance sports, Strength and Power sports and Muscular endurance sports, report a higher degree of pharmaceutical drug use in training situations (OOC) compared to during competitions (IC). This should be investigated further.

The relative high prevalence of pain killers (NSAIDs, M01 A and paracetamol, N02 B) is comparable to what has been reported by other studies (15, 49). Nonsteroidal anti-inflammatory drugs are amongst others used by athletes to treat acute muscle- and tendon injuries and muscle soreness, which are typical conditions related to exercise and physical performance. It is therefore not surprising that these substances are used extensively in sports, although indicators of overuse exist (21, 50). One challenge is that these pharmaceuticals are often available as Over-The-Counter making it difficult for the athletés doctor to control what products and how much is used. Another aspect to consider is that NSAIDs cause deterioration in respiratory function in approximately 10% of adults with asthma (51). It does not appear that the inappropriateness of using NSAIDs for patients diagnosed with asthma is well-known amongst the athletes, as the use of NSAIDs in combination with anti-asthmatics from either ATC group R03A and/or R03B was detected in 9.1% of the DCFs reporting NSAIDs use.

Tramadol (N02 A X02) will be listed on the WADA Prohibited List from 1st of January 2024.The result from the present study suggest that tramadol use is limited, as only 24 of 10 418 DCFs (0.2%) contain information about the substance. It is thus no reason to suspect inappropriate use of this substance amongst Norwegian athletes, at least for the period of the data collection.

Among the most surprising findings in the present study were the relatively common use of hypnotics (reported by ice hockey players on 8.2% of all DCFs) and alpine skiers (8.0% of all DCFs). As these pharmaceuticals are potentially addictive, it is worrying that they are used regularly by relatively young, healthy athletes. In ice hockey, its prevalent use may be due to a tight match schedule, late matches, while extensive travelling across time zones throughout the year and consequently lack of sleep may be an explanation among alpine skiers. This should be examined more carefully in future studies. Use of hypnotics in sport in ATC class N05C is to the best of our knowledge not discussed in the literature. However, the use of benzodiazepines in sport is recently covered (52). These substances, found in ATC class N05B, were only mentioned on 0.3% on the DCFs in the present study.

The use of antibiotics does appear to be less common among Norwegian athletes compared to studies from other countries (8, 9, 22, 37). Only 1.0% of the DCFs contained information of use of the most common oral antibiotics (J01 C, penicillin). The use of antibiotics in Norway is well controlled, and the strategy of the Norwegian government is to be one of the three European countries that uses the least antibiotics in humans (53).

Information about pharmaceutical usage amongst the general population in Norway, as reported in the Norwegian Prescription Database (NorPD) (35) revealed that use of the corticosteroid asthmatics (R03B) was slightly increasing in Defined Daily Dose/Thousand Inhabitants per Day (DDD/TID) in the period 2015–2019, but the prevalence was steady. The adrenergic inhalants (R03A) had an increase of almost 10% in DDD/TID. The anti-inflammatory painkillers (M01A) were unchanged whereas other analgesics and antipyretics (N02B) were increasing by almost 20% in DDD/TID and 35% in number of individuals, indicating that in addition to more usage, people are getting more on prescriptions. It was practically no change in use of hypnotics (N05C) in the general population.

### Strengths and limitations of the method

Using information about self-declared use of pharmaceuticals from DCFs is nothing new and has been used in several other studies (10, 54, 55). However, in the present study, the ATC system was used for classification of the pharmaceuticals. Few other studies have used the ATC system for classification of pharmaceuticals in sport (56), and none with comparable amount of data. By using ATC for classification, data for pharmaceuticals down to substance level (fifth level) or pharmacological or therapeutic subgroup (third level) the database will be a valuable source for information on specific substances or subgroups for e.g., anti-doping educators, sports physicians, and researchers.

The number of DCFs containing self-declared use of pharmaceuticals is the variable mainly used in this paper. A DCF with several pharmaceuticals could indicate anything from liberal use of pharmaceuticals to a period of health problems. Also, underreporting of the use of pharmaceuticals and supplements is a possible source of error in the data material. For many athletes, the doping control itself can be a stressful situation that leads to forgetfulness when, for example, it concerns which products they have used in the last seven days before the control. It is possible for the athlete to submit information about this to ADNO after the control. However, such post-registration of pharmaceuticals and supplement were not included in the present material, and there are no statistics on the extent to which this occurs. Another reason for underreporting may be that the athlete wants to hide the use of certain products. However, if it is related to prohibited substances, the use would be revealed in the urine sample anyway. The athlete therefore has everything to gain by being honest in the first instance. Another potential source of error is indistinct handwriting or misspelling of the products. With the paper based DCFs used during the study period, the handwriting of the athlete was crucial for the interpretation of the content. However, pharmacy students were doing the registration of the data, as they are skilled to interpret both pharmaceutical names and different handwritings. When in doubt, they discussed internally or with other members of the project group. Thus, the incorrect registration of the products as a source of error was then minimized.

Because of the fully anonymized database, DCFs from athletes being tested multiple times could not be merged. They often register the same pharmaceutical(s) on the DCFs of consecutive doping controls. Numbers and statistics in this paper refer to DCFs as a unit unless stated otherwise. As some athletes were tested more than once during the time-period, their data may disproportionally affect the mean values of the data material. This is particularly evident in sports with relative few athletes, where some athletes with a high use of prescription drugs and/or dietary supplements may affect the mean values of the sport/sport discipline.

## Conclusion

The ATC classification system works well for the mapping of pharmaceuticals in the DCFs. Using the DCFs as a tool in the process of mapping and understanding the athletes' use of pharmaceuticals needs to be further developed. The data obtained from this study is expected to be useful for athletes and supporting personnel like physicians, physical therapists, and nutritionists as well as for sport federations. It also indicates some areas to be explored further and understood better, like the culture for use of pharmaceuticals in sports disciplines with seemingly high or unusual usage patterns.

## Data Availability

The raw data supporting the conclusions of this article will be made available by the authors, without undue reservation.
